# The Jubilee Institute for Nurses

**Published:** 1891-03-28

**Authors:** 


### Passing Topics.

### THE JUBILEE INSTITUTE FOR NURSES.

The report of the Council of "Queen Victoria's Jubilee Institute for Nurses," which that body is privileged to submit to no less august a personage than the Queen herself, is a portentous but unsatisfying document. Its compilers appear content to supply a record of a minimum of good work accomplished, garnished with indications of better things to come.The report is, in fact, rich in the suggestion of possibilities, but shows a poverty of performance not wholly comprehensible.

We should prefer, to believe that the modesty of the Council has prevented them from doing full justice to themselves, and that during the period embraced their activity has been greater than the report makes manifest. Still we cannot refuse to accept, in the exact terms in which it is offered, their deliberate and formal statement, and as we possess no other means of knowledge, the observations which occur to us are based wholly upon the information the Council have published to the world.

No one will deny that the field lying before the Institute is practically unlimited, or that the conditions under which the Society was founded gave it at the very outset, the distinct advantages of a great prestige and material means, which, if inadequate to the ultimate needs, were not insignificant. We are told that there have been frequent meetings of the Council, and that various committees have been formed to facilitate the general work, but when we look for the results of all these deliberations, we must confess to a feeling of disappointment. One would have thought that after the passage of a period exceeding two years the preliminaries of organization would have fallen into the background, and that there would have been something within reach more substantial than an outline of merely prospective undertakings. True it is, that in the otherwise shadowy picture presented to us, one corner of the canvas is occupied by a parade of eleven trained nurses and five probationers, but when we are told that the effective members of this not very imposing array—the product of two or three years of organization—have been distributed over an area wide enough to include the metropolis, Dublin, Bolton, and Little Haven, we are compelled to inquire what the Council expect of these attenuated forces?

We do not complain of the attempt to give a national scope to a work set on foot by a national subscription, but we should have better liked it had the attempt been more seriously made. Eleven nurses is scarcely an adequate muster wherewith to occupy England, Ireland, and Wales, while the omission of Scotland is calculated to raise a grievance over the border.

We read that the number of "Queen's Nurses" is now 94, and, of course, if this body were engaged in actively and continuously working for the Institute, or if anything approaching that number were in training, we should regard it as a very solid and satisfactory achievement. But as we understand the matter, this band of 94, or the large majority of them, existed as trained nurses before the Jubilee Institute was formed or thought of, and all that has been done has been to select these particular nurses as qualified for the honour of enrolment in the ranks of the "Queen's Nurses," while they continue to occupy the same positions, and to discharge the same duties as heretofore, in respect of the several hospitals or other institutions to which they are attached.

Their connection with the Institute is therefore little more than nominal, and we fail to appreciate the advantage of this distribution of purely honorary distinctions or to perceive how it promotes the primary function of the Institution—the provision of nurses to tend the sick poor in their own homes. "We consider," says the Council, "our chief work to be the training of nurses"; yet the most careful search among the paragraphs of this report fails to show that any nurses have been trained or are in course of training beyond the sixteen referred to. Is this really the total of the training work accomplished by the Jubilee Institution? Agricola.

